# Phage Therapy: An Alternative Approach to Combating Multidrug-Resistant Bacterial Infections in Cystic Fibrosis

**DOI:** 10.3390/ijms25158321

**Published:** 2024-07-30

**Authors:** Mario Cocorullo, Giovanni Stelitano, Laurent Robert Chiarelli

**Affiliations:** Department of Biology and Biotechnology “Lazzaro Spallanzani”, University of Pavia, Via A. Ferrata 9, 27100 Pavia, Italy; mario.cocorullo01@universitadipavia.it (M.C.); giovanni.stelitano@unipv.it (G.S.)

**Keywords:** cystic fibrosis, bacterial infection, antimicrobial resistance, alternative strategies, phage therapy

## Abstract

Patients with cystic fibrosis (CF) are prone to developing life-threatening lung infections with a variety of pathogens that are difficult to eradicate, such as *Burkholderia cepacia complex (Bcc)*, *Hemophilus influenzae*, *Mycobacterium abscessus* (*Mab*), *Pseudomonas aeruginosa*, and *Staphylococcus aureus*. These infections still remain an important issue, despite the therapy for CF having considerably improved in recent years. Moreover, prolonged exposure to antibiotics in combination favors the development and spread of multi-resistant bacteria; thus, the development of alternative strategies is crucial to counter antimicrobial resistance. In this context, phage therapy, i.e., the use of phages, viruses that specifically infect bacteria, has become a promising strategy. In this review, we aim to address the current status of phage therapy in the management of multidrug-resistant infections, from compassionate use cases to ongoing clinical trials, as well as the challenges this approach presents in the particular context of CF patients.

## 1. Cystic Fibrosis and Related Bacterial Infections

Cystic fibrosis (CF) is an autosomal recessive disease caused by mutations in the *ctrl* gene leading a misfunction in the CF transmembrane conductance regulator (CFTR), a cAMP-regulated chloride channel expressed in epithelial cells of different tissues [1]. The loss of ion regulation involves the accumulation of a thick mucus over the lungs, causing an insufficient mucociliary clearance as well as and alteration in phagocytosis. This condition gives rise to a suitable environment for the colonization of different bacteria and fungi, which often establish persistent infections [2,3]. In particular, bacterial species that may colonize this mucus include *Burkholderia cepacia complex (Bcc)*, *Hemophilus influenzae*, *Mycobacterium abscessus (Mab)*, *Pseudomonas aeruginosa*, and *Staphylococcus aureus*. Among the fungi *Aspergillus fumigatus*, *Penicillium sppm.* and *Candida albicans* are the most common, sometimes even establishing dual-species biofilms as reported in some pediatric cases for *C. albicans* and *P. aeruginosa* [3,4].

Furthermore, as the stagnation of mucus inside the lungs leads to chronic inflammation and a general decrease in innate defenses, these conditions tend to worsen over time, leading to the onset of respiratory failure [1,5].

The severity of the disease may depend on a combination of genetic and environmental factors [6]. More than 2000 CFTR gene mutations have been reported so far, associated with more or less serious forms of CF [7,8]. In addition, different genes, called modifiers, have been identified able to interact positively or negatively with the CFTR [9,10]. Moreover, environmental factors as type of treatments, the adherence to the therapy and the lifestyle adopted can significantly influence the evolution of the disease. Anyway, although the severity of the disease may vary considerably from person to person, the progressive impairment of lung tissue caused by persistent infection and inflammation remain the main cause of morbidity pwCF. In any case, in recent years the therapy for CF has considerably been improved, especially following the introduction of so-called CTFR modulators, drugs that can restore the function of the CFTR protein of about 40–50% [11].

CTFR modulators are drugs that can improve or restore the expression, the function, or the stability of a malfunctioning CFTR. Based on the effects they exert on CFTR mutations, they are classified into five groups: potentiators, correctors, stabilizers, read-through agents, and amplifiers [8].

The potentiators are molecules that increase the probability of channel opening in CTFRs with mutations leading to impaired channel gating; these mutations are present in about 5% of pwCF [12]. Correctors can rescue folding or the processing of CFTRs carrying mutations that cause a mistrafficking of the protein to the plasma membrane. This is the case with the F508del mutation, the most prevalent in pwCF [12]. Stabilizer increases the stability of CFTR proteins with mutations that significantly reduce the half-life. The read-through agents are compounds that can induce a ribosomal the over-reading of premature termination codon, allowing for the introduction of an amino acid instead, thus permitting the translation to continue. Therefore, these compounds can rescue mutations that can introduce a termination codon such as nonsense, frameshift, or splicing variants. Finally, amplifiers increase expression of the mRNA coding for the CTFR, thus the production of the protein. These compounds are useful for mutations leading to reduced synthesis or maturation of the protein [12]. An example is cycloheximide, which restored mature CFTR expression, by correcting mutations such as A455E, S492F, ΔI507, and R560T [12].

Nevertheless, although this innovative therapy is improving the lives of several patients with CF (pwCF), these type of drugs are not yet available to treat all known genetic variants, and opportunistic infections still remain an important issue [13]. With this review, we aim to draw a general picture of the most important CF bacterial pathogens, focusing on the alternative treatment of their related infections with bacteriophages.

## 2. An Overview on Main Pathogens of Patients with CF

As mentioned, among the bacterial pathogens that generally infects pwCF, *Bcc*, *H. influenzae*, *Mab*, *P. aeruginosa*, and *S. aureus* are the main ones [13,14,15].

In general, the infections of the abovementioned bacteria are difficult to treat since their intrinsic and acquired resistance to antibiotic or different strategies to avoid the host’s immune system.

For example, the thick cell wall of *M. abscessus*, which is typical of mycobacterial species, as well as the cell wall of Gram-negatives *P. aeruginosa* and *B. cenocepacia*, may impair the crossing of many antibiotics, as can the efflux pumps that are able of exporting various molecules toxic for these microorganisms [16].

Moreover, the natural antimicrobial resistance of bacteria is combined to their attitude to establish biofilms [2], communities of the same or different species impenetrable to most drugs [16]. These structures are composed by a matrix of autogenic extracellular polymers, such as polysaccharides, proteins, nucleic acids, and lipids, that surround the bacteria cells and characterized by a specific elasticity resistant to mechanical stress. This matrix is causative of the improvement of the antibiotic resistance of the bacterial community of even thousand times [17].

Specifically, *Bcc* is composed by around 26 species, of which *B. cenocepacia* and *B. multivorans* are the most common [18]. Among all the species of this complex, most of them may cause severe or deathly infections in pwCF, which are hard to eradicate with antibiotics, giving low priority for lung transplantation to infected pwCF [19].

*H. influenzae* is a Gram-negative pathobiont classified in six capsulated serotypes and non-capsulated strains [20]. Overall, the serotype B capsulated strain is the most infective, but an effective vaccine is available (Hib). Recently, pathogenic non-typeable serotypes are emerging and they already became a global concern, mostly in America and Europe [20].

*M. abscessus* (*Mab*) is a nontuberculous mycobacterium and an emerging pathogen responsible of severe infections particularly in people suffering from congenital lung diseases, including pwCF. This opportunistic pathogen is gaining increased importance since its incidence in industrialized countries is even higher compared to *M. tuberculosis* [21]. *Mab* may establish chronic infections in immunocompromised patients and people with respiratory diseases and may be causative of complications after pulmonary transplant [22,23,24]. It can be found in a smooth variant rich in glycopeptidolipids and associated with biofilm or in a more virulent rough variant poor in glycopeptidolipids and related to persistent and chronic infections [25,26,27]. As previously stated, *Mab* is intrinsically resistant to many antibiotics since it possesses a high number of efflux pumps and a thick cell wall, peculiar of mycobacteria species, and even thicker than Gram-positive bacteria [16,21]. Indeed, treatments against this opportunistic pathogen are mainly based on repurposing drugs developed for therapeutic regimens against other mycobacteria such as *M. tuberculosis* and *M. avium* [28]. Moreover, currently, a standard drug regimen to treat *Mab* infections is not established, although consensus recommendations have been published by the US Cystic Fibrosis Foundation and the European Cystic Fibrosis Society [29]. So, the high difficulty in fighting and eradicating *Mab* infections is pushing researchers to develop novel anti-*Mab* strategies and specific drugs.

*P. aeruginosa* is, today, one of the most studied microorganisms and one the main pathogens of pwCF [15]. Its plastic genome, featured by numerous virulence factors both intrinsic and acquired, confers to this bacterium a high adaptability to the environment, such as the thick mucus of pwCF [30]. Commonly, this plasticity is also causative of *P. aeruginosa* multidrug resistance, such as resistance to aminoglycosides, cephalosporins, fluoroquinolones, and carbapenems [31]. Finally, it may also establish biofilms in chronic infections, exacerbating the clinical pictures of patients [32].

Finally, *S. aureus* is a Gram-positive bacterium and an opportunistic superbug belonging to ESKAPEs [33]. Its genomic plasticity was causative of the acquisition of different virulence factors and drug resistances, so that multidrug-resistant *S. aureus* and hypervirulence strains are nowadays a challenging concern for human health [34]. For example, the widespread methicillin-resistant *S. aureus* (MRSA) may cause infections in skin and soft tissues as well as endocarditis, bacteremia, and osteomyelitis [14,35].

Considering the difficulties in treating these bacterial infections, for the reasons outlined above, as well as the peculiar presence of thick mucus at the site of infection that impairs the immune system and antibiotics from reaching targets, alternative or complementary therapies are required to eradicate the infection of these pathogens in pwCF.

Indeed, novel strategies are under development to fight multidrug-resistant strains as well as chronic infections in pwCF [36]. Among these anti-virulence compounds like quorum sensing inhibitors and iron chelators, the use of nitric oxide or bacteriophages have been successfully exploited to treat some infections of *M. abscessus*, *S. aureus*, and *P. aeruginosa* in pwCF [16,37,38,39,40].

The use of bacteriophages in therapy is not something new. It has been largely exploited in eastern Europe for decades and more recently it caught the attention of western Europe [41] so that new treatments are under development against persistent infections, in particular in pwCF and fragile individuals [14].

## 3. Phage Therapy Overall

Since the 1940s, the use of modern antibiotics as antimicrobial therapeutics in the Western world has largely replaced the administration of phages. However, recently, the use of phage as supportive therapy to systemic antibiotics for the treatment of complex infections, known as “phage therapy”, has gained interest considering the increasing burden of antimicrobial resistance (AMR), multi-resistant bacteria (MDR), and the stalled development of new antibiotics. This is confirmed by the numerous published cases series and case reports of the use of bacteriophages for the treatment of bacterial infections [42,43].

Bacteriophages have been considered for therapeutic use for almost a century, since they were first identified as viruses that attack bacterial targets, and, due to the successes reported by the end of the First World War, phage therapy became widespread in eastern Europe and the Soviet Union [44]. Although the first antibiotics, penicillin and streptomycin, became widely available in the early 40s and were adopted in the western Europe, phage therapy continued to be used for many years in the eastern Europe. For instance, published cases of clinical trials, performed in Poland and Georgia, have shown promising results due to the different mechanism of phages compared to the antibiotics. These trials mainly involved patients with associated infection from *Pseudomonas aeruginosa* or *Klebsiella pneumoniae*, nosocomial bacteria well-known for their antibiotic resistance [44,45,46].

Phages are a class of viruses that infect bacteria in order to complete their own life cycle. After having introduced the genetic material into their host, phages exploit bacterial metabolic functions to either enter a lysogenic state, also called temperate phages, or follow a strictly lytic life cycle. During the lysogenic cycle, phages integrate their genome into the bacterial chromosome, called prophage, so it is replicated together with DNA of the bacterial host [42]. During this phase, they are mostly dormant, but can anyway modify gene expressions, and they can be activated by many different signals to the lytic state. On the contrary, lytic phages cannot enter in a lysogenic state, but after having infected the host organism, the latter is rapidly killed by the lysis and the release of the subsequent generation of biologically infectious viruses [42].

Phages are widely distributed in the natural environment and have considerable value for clinical use. Therapeutic phages are usually lytic and recognize and bind to specific bacteria, mostly through specific receptors of the external membrane of the host [37,47]. They offer many different advantages compared to antibiotic treatments, such as shorter time of treatment and a high specificity, although their larger size may restrict the penetration into bacterial replication sites. Moreover, they have the unique ability to replicate at the sites of infection, and usually have low toxicity, so they can be used in combination as a phage cocktail to better fight the target organisms [37,47].

The characteristics of an ideal phage for phage therapy are typically described as obligately lytic hence virulent, with a broad and also species-specific host range. Notably, phage host range is primarily determined by recognition of bacterial cell surface features by the phage tail fiber, and the modification of these structures should be able to increase phage host range for therapeutic applications [48].

In the choice of phages for clinical use, it is important to consider host specificity. This condition prevents the lysis of bacteria that should not be targeted with the subsequent effect that can arise. However, it is also important to be aware of the potential development of resistance mechanisms in bacteria due to continuous phage applications, which can lead to co-evolution. Several aspects are involved in phage resistance in vivo, and some evaluations in vitro have raised the possibility of the development of phage resistance [49]. Bacteria can evolve many antiviral strategies by interfering with the phage life cycle and acquiring chromosomal mutation, which can hijack the bacteriophage from finding their receptors on the bacterial surface [50]. Although phage resistance is a rare event, this problem has been reported in some clinical trials, leading to the need to modify the composition of the phage cocktail for treatment regimens in these subjects [51,52].

To enhance the effectiveness of phages and address phage resistance, several approaches have been adopted, for instance, phage cocktails or combinations of phage and antimicrobial drugs. The use of phages offers several benefits like their self-replicating capacity, which allows for them to replicate easily; moreover, they are also much cheaper and faster to prepare in contrast with antibiotics. Further, they are pretty specific, and no phage replication occurs in human tissues, supporting the observation of remarkable safety in reported cases of treatments [53,54].

The different specificity of these phages depends on their classification. Phages can be roughly divided into monovalent (with a narrow host range) and polyvalent (with a broad host range). Nevertheless, researchers often describe phages differently, and there is still much inconsistency in the use of these terms. Significant investigation has been carried out into the use of both narrow- and broad-host range phages in the treatment of infections and diseases caused by MDR bacteria. The effectiveness of phage therapy is therefore determined by the host range of these phages [55].

It is noteworthy that phage therapy can “re-sensitize” previously antibiotic-resistant bacteria to antibiotics in vitro, a concept known as “phage steering” [56]. However, it has not yet been demonstrated that phage steering can properly work against an infection. Therefore, phage steering is a therapeutic strategy that utilizes phages to kill phage-sensitive bacteria while directing the bacteria that survived to acquire an antibiotic-sensitivity. Actually, this therapy seems able to improve bacterial clearance through antibiotic re-sensitization in association with phage resistance [56].

The whole idea of phage steering is to obtain phage resistance and subsequent susceptibility to antibiotics. In the study of Ashworth et al., phage resistance developed in the in vivo model, but also in the lungs, even in the absence of phage treatment. The lung was the only tissue to exhibit near-total resistance to the incoming phages, while other tissues showed varying levels of phage resistance in the absence of phage treatment. The final point of this approach is that phage steering involves the use of bacteriophages to target and eliminate most phage-sensitive bacteria, while also promoting the development of an antibiotic-sensitive phenotype in the remaining population [56].

It is often believed that phages that replicate using the lytic life cycle are the only ones that have therapeutic value, as the only result of infection is the lysis of the host bacterial cell and release of progeny phages. On the other hand, temperate phages are generally considered somewhat unfavorable because a high proportion of the infected bacterial cells persist to become lysogens and carry the virus as a prophage [57]. It has been observed that temperate phages can be engineered to suppress the lysogenic state by removing part or all of the repressor gene. That is important, since also if the better choice is still the use of lytic phages, for some pathogens, there is not enough availability of this kind of phage; hence, the interest will move toward the available temperate phages, which can be improved by the lysogenic state silencing by removing part or all of the repressor gene [57]. For instance, bacteriophages for *Burkholderia* spp. are mainly temperate phages [58], limiting their use against these important pathogens of pwCF. However, temperate phages can represent a useful platform for engineering a phage displaying a higher virulence and broader host range, as in the case of the phage Milagro [49].

However, lysogenic phage can still produce lysis and be virulent, and not all lysogenic phages are able to form stable lysogens, so it is being debated if they may have therapeutic utility. Moreover, the formation of the lysogens can also depend on external factors, such as the host and the environment [59]. In this context, Lauman and Dennis developed a set of metrics to evaluate eight specific phages of *Burkholderia* spp. to determine the tendency to form stable lysogens and their antibacterial activity, in order to assess their potential therapeutic role. This approach should be useful to also exploit temperate phages in a polyphage cocktail [60]. The various recent studies moving in this direction are indeed beginning to lay important foundations for the isolation and development of new phages for use in phage therapy [49,60,61,62].

Typically, bacteria lysogenized with a phage are resistant to the infection by a phage of the same type, a phenomenon called superinfection exclusion. However, under particular stress conditions, superinfective phages, with the ability the successfully infect lysogenized-bacteria, can emerge [63]. This observation led to the idea of engineering phages, with a view to obtaining superinfectious variants. For example, Prokopczuk and co-workers realized an engineered superinfective version of the *P. aeruginosa* filamentous phage Pf4, which was successfully tested in a burn wound infection model, confirming the potential of this approach [64].

Bacteria and phages have a co-evolutionary relationship, where bacteria develop mechanisms to resist phage infection, and phages adapt to overcome those bacterial defenses. The emergence of phage-resistant bacteria presents a challenge for phage therapy. However, phages have the potential to be pre-adapted to their target bacteria, both in vitro and in vivo. Phage pre-adaptation involves a process of training phages in prior in vitro antagonistic evolution [53]. Phage therapy, which has been a long-standing practice since the beginning of 20th century, has undergone a strong revival in the last years, after being largely abandoned in Western countries for many years. A growing number of clinical trials are underway to test the role of various phage products in the fight against multidrug infections. It is intriguing to note that clinical trials of recombinant and synthetic phages are now being initiated but are subject to greater scrutiny in terms of safety. Although there is a lack of efficacy data from clinical trials, several countries have implemented a “parallel track” for the approval of phage therapy for individual cases of phage therapy on a supportive care basis when antibiotic options have failed [65].

Synthetic biology, which combines the principles of biology and engineering, can be exploited to design existing biological agents to perform tasks that do not occur naturally. There is clear and remarkable potential in therapeutic phage bioengineering, with innovative approaches being tested on a continuous scale [66]. The results obtained, for instance, with the administration of mycobacteriophage against *M. abscessus* subsp. *massiliense* in a zebrafish model of CF, allowed for evaluating the influence generated by innate immunity, since in embryos lacking macrophages, phage therapy was not as effective [67]. In vitro assessment of phage therapy on *P. aeruginosa* clinical strains showed great biofilm reductions, compared with the result obtained from the same strains in planktonic form, where the same treatment was not active [68]. Some studies underlined the host-specificity of the phages, observing that many of them can interfere with one or a few strains of the same bacterium, for instance, of *P. aeruginosa*, but not with all strains. Nonetheless this paves the way for the possibility to obtain several phages attacking the same strain, further obtaining the possibility to modify the phage cocktail in case of resistance [69].

Improving the knowledge on phage–host and phage–human interactions, phage dynamics and genome function are fundamental to enable the generation of a new strategy to combat bacterial infections and to face the challenges related to phage therapy [70].

## 4. Phage Therapy in Patients with Cystic Fibrosis

Although the introduction of CFTR modulators has improved lung function in many pwCF, these innovative drugs cannot be used to treat all mutations, and benefits against inflammation and infection still remain limited. Bacterial infections therefore remain a major challenge. Moreover, infections with pathogens such as *B. cenocepacia* or *M. abscessus* are particularly difficult to eradicate and often preclude lung transplantation [71].

The increasing spread of antibiotic-resistant bacteria has revived the use bacteriophages to treat infections. Indeed, phage therapy may have several advantages with respect to conventional antimicrobials, for instance, a high specificity for the bacterial host, which limits off-target effects, as well as the killing of commensal bacteria, lower toxicity, and lower manufacturing costs [72,73,74,75].

**Figure 1:**
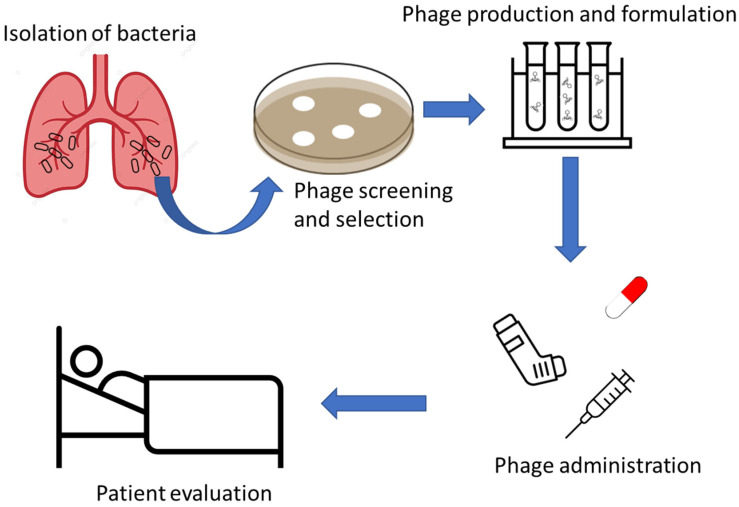
Outline of the approach mainly used for phage therapy of lung infections.

Phages have been recently used in several cases as compassionate therapy to treat drug-resistant bacterial infections [75,76,77], including infections in pwCF caused by *Pseudomonas aeruginosa* [38,78], *Staphylococcus aureus* [38], *Achromobacter* [79,80,81,82], *Burkholderia multivorans* [83], and *Burkholderia dolosa* [84], as well as *Mycobacterium abscessus* [37,73,85,86]. These cases demonstrated the potential of this approach for the treatment of lung infections resistant to all conventional therapies, as well as the safety of phage therapy, also supported by the positive outcome in some of them. Figure 1 schematizes the main approach used in these studies: the pathogen is isolated, e.g., from patient sputum, and tested for susceptibility to an appropriate panel or library of phages. The most suitable phages are selected and evaluated for their efficacy, then formulated for the appropriate route of administration (intravenous, aerosol, oral, etc.). Treated patients are then evaluated for adverse events and clinical outcome (Figure 1).

For instance, interesting information emerged from the compassionate use of phages in 20 pwCF with *M. abscessus* infection [37]. In this study, personalized phage regimens were administered, and to this end, mycobacterial isolates from individual patients were assayed against a panel of about 25 phages, known to infect *Mab* or *M. tuberculosis*. The phage treatment was well tolerated, and no phage resistance occurred, even in cases where a single phage was administered. Moreover, half of the patients showed a favorable response; in some cases, complete resolution of the infection; and one successfully underwent a lung transplant. However, for some patients, the clinical benefit was rather low; some patients developed neutralizing antibodies, which may have contributed to the low response, although the strong neutralization toward one phage in two patients did not prevent a favorable outcome, so the reason for the variability in response is still unclear [37].

It is anyway worth noting that *M. abscessus* lineages are characterized by relatively high genetic stability, which could limit the emergence of phage-resistant strains [86].

By contrast, *P. aeruginosa* shows high genetic plasticity and hypermutator genotypes [87], favoring the emergence of phage resistance [88]. This has been reported, for instance, in a recent case report describing the individualized use of phage therapy in two pwCF with *P. aeruginosa* infection [89]. Also in the study, *P. aeruginosa* isolated from the patients were tested against library of phage with lytic activity against *P. aeruginosa*. Both patients displayed symptomatic improvement after the treatment, but at the end of therapy had a regrowth of *P. aeruginosa*, conceivably due to the emergence of phage resistance [89]. This does not exclude that cases of *P. aeruginosa* infection can be successfully treated by phage therapy, as described in a case report in which a pwCF with chronic *P. aeruginosa* infection was successfully treated, enabling the patient to undergo lung transplantation [78]. Indeed, this patient, who was intravenously administered the four-bacteriophage cocktail AB-PAO [84], had a positive outcome, with no related adverse events, and no recurrent pneumonia within 100 days of follow-up [78].

Besides the treatment of pwCF with mycobacterial or pseudomonal infections, cases of compassionate treatment with phages in patients with infection with *Burkholderia* spp. [83,84] and *Achromobacter* spp. infections [55,80,81,82] have also been reported. Even in this type of infection, however, the effects of phage therapy, as well as the outcome of patients, proved to be very heterogeneous. For instance, the study of Aslam and co-workers [84] reports a pwCF with *Burkholderia dolosa* infection, who received antibiotic treatment combined with the administration of a single phage with in vitro activity against *B dolosa*. The patients experienced no related adverse events, and general improvement, with decreased bacterial load, but developed severe septicaemia from *B. dolosa* on week 10 of phage therapy [84]. Unfortunately, a similar outcome was described by Haidar et al. for a pwCF with multidrug-resistant *Burkholderia multivorans* infection [83]. By contrast, in two case reports the phage treatment of *Achromobacter* species infection in pwCF resulted in a positive outcome [80,81].

It must always be taken into account that these cases of compassionate use of phages are characterized by high heterogeneity regarding the pathogens involved, the severity of the disease, and the overall condition of the patients, and often lacks standardization and a control group, so conclusions must still be taken with caution [90].

However, the potential of phage therapy for the treatment of lung infection in a particular context such as in pwFC has yet to be fully explored, and for several questions, such as the administration route, length of the treatment, and the concomitant administration of mucolytic agents, the formulations have yet to be solved.

For example, the altered mucociliary clearance, and the production of dense, thick mucus, favors the establishment of microbes and affects immune responses to infection and inflammation. Moreover, in this type of environment, bacteria tend to form biofilms.

It is clear that this peculiar environment can have a strong impact on the efficacy of drugs, especially when administered by inhalation. For example, it has been seen that high mucin and DNA levels increase the viscosity of CF dysfunctional mucus, limiting the diffusion of antibiotics [91,92]. The same problem could conceivably occur with phage administration.

For instance, a study on the compassionate use of phages, in a series of patients with mycobacterial lung infections showed that intravenous administration was more effective in treating disseminated infections, particularly in the presence of structural damage to the lungs or dysfunctional mucus. In contrast, administration by nebulization limited neutralization of the phages by the immune system of the patients [37].

As mentioned above, CF pathogens tend to form a biofilm which, due to its complex composition, can hinder the diffusion of antibiotics, making treatment even more difficult. However, some bacteriophages produce depolymerases, enzymes that are capable of modifying the polysaccharides involved in the biofilm structure, thus improving their penetration into the matrix and their effectiveness [93]. Together with the aforementioned phage steering, this evidence supports the potential of combining phage therapy with conventional antibiotics for the treatment of multidrug-resistant infections, as confirmed by several in vitro and in vivo studies [94,95,96,97,98].

A further problem could arise from the fact that some pathogens, such as *M. abscessus*, are intracellular, implying the need for phages to reach bacteria residing in human cells. Indeed, the absence of specific receptors on the surface of mammalian cells would preclude phage internalization, suggesting that phage therapy could only be effective with extracellular bacteria [14]. Furthermore, *M. abscessus* usually resides in macrophages, so there is also the possibility of phage clearance from the intracellular environment [99]. However, different recent studies reported the possibility of internalization of phages by phagocytic cells [100,101], as well as the possibility of phages penetrating mammalian cells and killing intracellular *M. abscessus* [14].

The restricted host range of phages could be another limitation for phage therapy, particularly in the context of cystic fibrosis, which is characterized by multiple infections. To circumvent this problem, several approaches are used, such as the use of phage mixtures, phage libraries, extensive screening, and genetic engineering techniques [102].

Currently, phage therapy for the treatment of infections in pwCFs has been used mainly in compassionate use cases, which, despite the lack of rigor and consistency in treatment and monitoring, have nevertheless provided a number of useful insights for the design of clinical trials. A multicenter clinical study was recently concluded (NCT04596319) [103]. This study was conducted to evaluate the safety, tolerability, pharmacokinetics, and pharmacodynamics of a multi-phage cocktail in pwCF with chronic *P. aeruginosa* lung infections, demonstrating the tolerability and efficacy of the formulation used [103]. Another recently concluded study (Cystic Fibrosis Bacteriophage Study at Yale CYPHY, NCT04684641), a phase 1/2 single-site, prospective, randomized, parallel, placebo-controlled, double-blinded trial, used Yale Phage Therapy 01 (YPT-01), a nebulized single-phage therapy [104]. However, although phage therapy in the study was safe, no differences in sputum in bacterial load or outcome were observed between the treated and placebo groups [105]. In addition, several clinical trials of phage therapy in CF infections by *P. aeruginosa* (NCT01818206, NCT04684641, NCT05453578, NCT05010577, and NCT04596319) or by nontuberculous mycobacteria (NCT06262282) are currently ongoing (Table 1) [105].

## 5. Conclusions

Although progress in research has dramatically improved the life of patients with cystic fibrosis, infections by multidrug-resistant bacteria continue to represent a major challenge. Among the alternative therapeutic strategies to overcome the lack of new effective antibiotics, phage therapy is standing out in particular. However, the peculiarities found in cystic fibrosis infections pose several difficulties to be overcome, leaving some questions still unanswered, such as the best route of administration, phage formulation and concomitant use of antibiotics or mucolytic agents, and the duration of the treatment period. However, the recent use of phage therapy in compassionate cases has provided encouraging indications of its efficacy, and several clinical trials currently underway will help implement this important approach in the cure of pwCF.

## Figures and Tables

**Table 1:** Currently ongoing or completed clinical trials of phage therapy in pwCF [105].

ClinicalTrials.gov ID	Official Title	Pathogen	Type of Phage(s)	Current Status	Last Update Posted
NCT04684641	CYstic Fibrosis bacterioPHage Study at Yale (CYPHY): A Single-site, Randomized, Double-blind, Placebo-controlled Study of Bacteriophage Therapy YPT-01 for Pseudomonas Aeruginosa Infections in Adults with Cystic Fibrosis	*P. aeruginosa*	Single phage	Completed	18 November 2023
NCT01818206	Bacteriophages Effects on Pseudomonas Aeruginosa Presents in Sputum of Cystic Fibrosis (CF) Patients	*P. aeruginosa*	Cocktail of 10 phages	Completed	5 September 2013
NCT05453578	A Phase 1b/2, Multi-Centered, Randomized, Double-Blind, Placebo-Controlled Trial of the Safety and Microbiological Activity of a Single Dose of Bacteriophage Therapy in Cystic Fibrosis Subjects Colonized with Pseudomonas Aeruginosa	*P. aeruginosa*	Cocktail of 4 phages	Recruiting	3 June 2024
NCT05010577	A Phase 1b/2a, Randomized, Double-Blind, Placebo-Controlled, Multicenter Study to Evaluate Nebulized Bacteriophage Treatment in Outpatient Adult Cystic Fibrosis (CF) Subjects with Chronic Pseudomonas Aeruginosa (PsA) Pulmonary Infection	*P. Aeruginosa*	Single phage	Active, not recruiting	18 October 2023
NCT06262282	A Prospective Standardized Assessment of People with Cystic Fibrosis and Non-tuberculosis Mycobacteria Pulmonary Disease Undergoing Treatment with Mycobacteriophage (POSTSTAMP)	Nontuberculous mycobacteria (NTM)		Enrolling by invitation	16 February 2024
NCT04596319	A Phase 1b/2a, Multi-Center, Double-Blind, Randomized, Placebo-Controlled, Single and Multiple Ascending Dose Study to Evaluate the Safety and Tolerability of AP-PA02 Multi-Phage Therapeutic Candidate for Inhalation in Subjects with Cystic Fibrosis and Chronic Pulmonary Pseudomonas Aeruginosa (Pa) Infection	*P. Aeruginosa*	Multi-phage cocktail	Completed	31 January 2024
